# Supplementary material for: Petascale pipeline for precise alignment of images from serial section electron microscopy
Source: Nat Commun. 2024 Jan 4;15:289. doi: 10.1038/s41467-023-44354-0 (PMC10767115; doi:10.1038/s41467-023-44354-0)
Supplement: Supplementary file 1 — Supplementary Figures [file 41467_2023_44354_MOESM1_ESM.pdf]

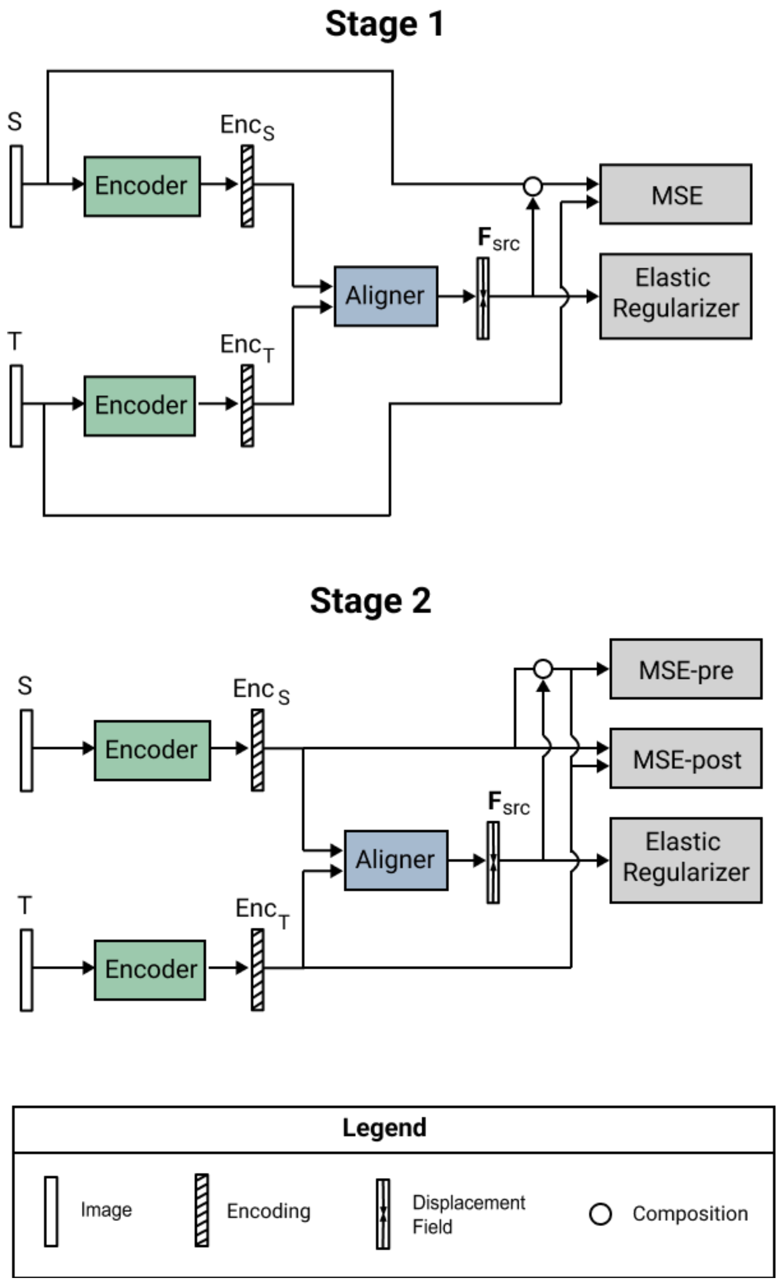

Figure S1. Schematic illustration of loss components during the training stages. During the first stage, loss is a weighted sum of MSE between images after alignment and elastic regularizer, both of which are being minimized. During the second stage, loss is formulated as a weighted sum of elastic regularizer, MSE between encodings after alignment (MSE-post), and MSE between encodings before alignment (MSE-pre). During the second stage, elastic regularizer and MSE-post are being minimized, while MSE-pre is being maximized. Loss is propagated through both the encoder and the aligner during both stages of training.

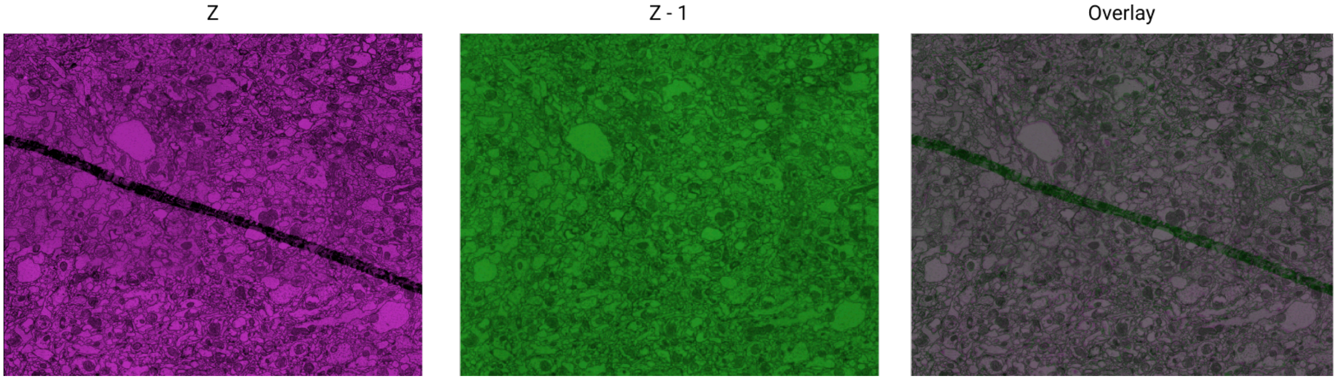

Figure S2. Pixel overlay of neighboring sections after v15 alignment. In the overlay, gray color corresponds to well aligned locations with similar intensity values for z and z-1. Width of each image is 30 μm.

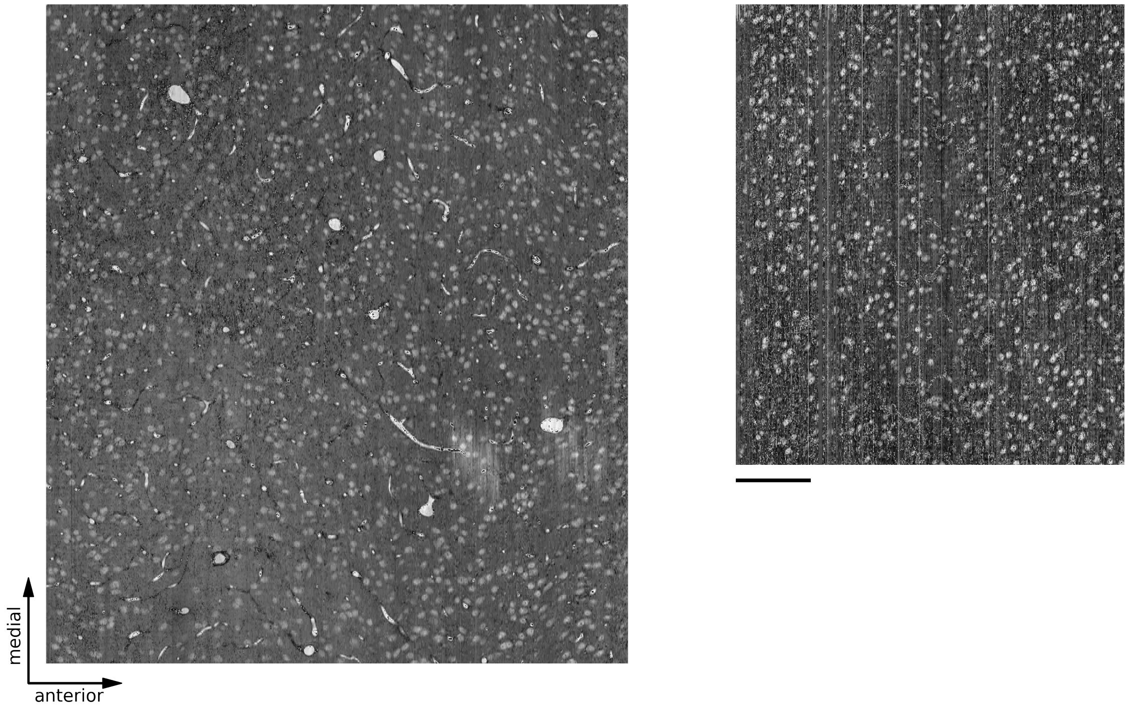

Figure S3. Alignment of mouse cortex datasets with over 10,000 sections. (**a**) A view of a cubic millimeter of mouse cortex with 20,000 sections after alignment by our preliminary pipeline, where each column represents a section of the data. (**b**) Similar view of another mouse cortex with 13,000 sections after alignment by our preliminary pipeline. Scale bar is 100 μm.

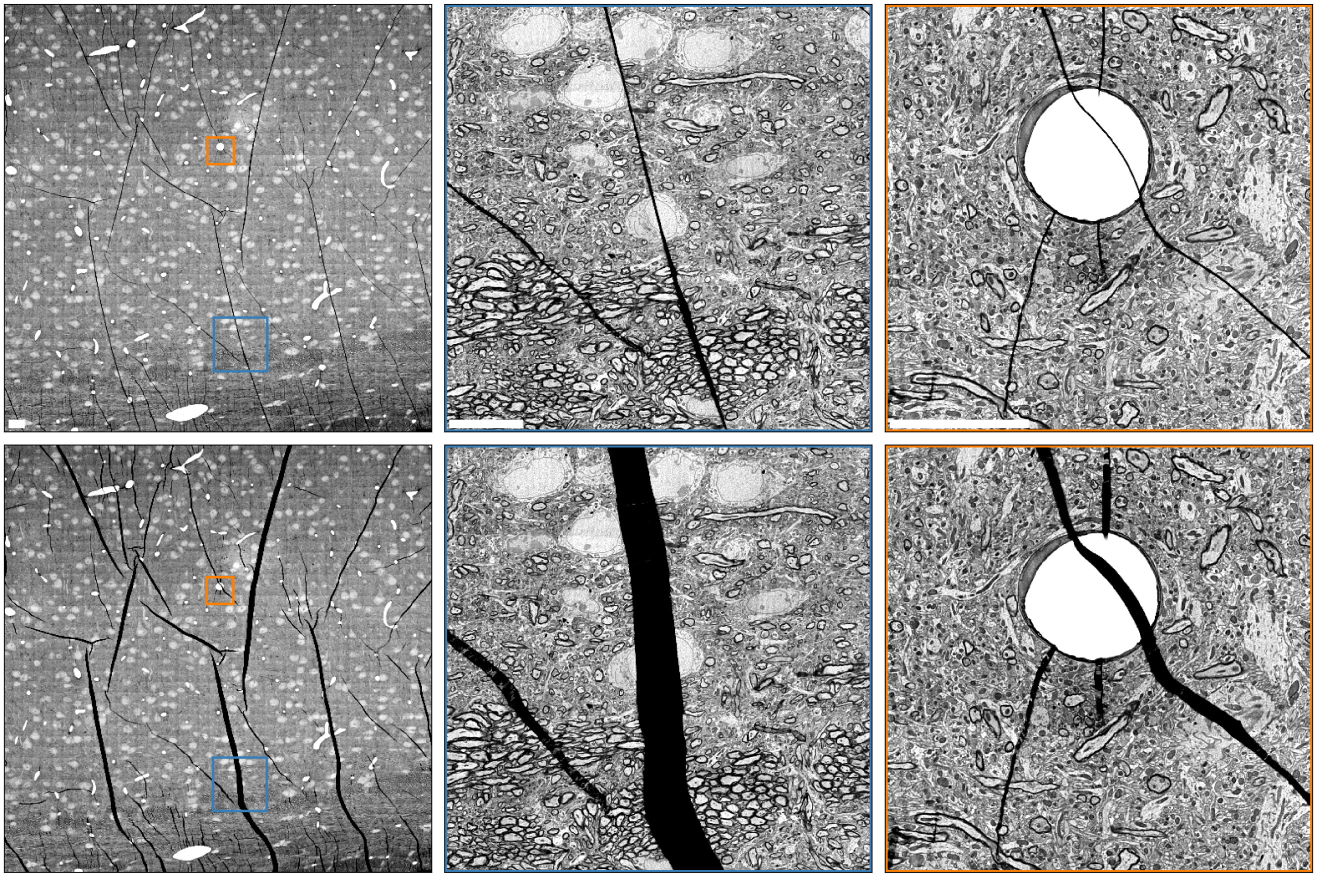

Figure S4. **Folds at multiple resolutions.** Single section views of mouse cortex data (Fig. 3a) before (**a**-**c**), and after (**d**-**f**) applying our alignment pipeline, with blue and orange insets displayed at higher resolution. Scale bars in (a-c) are 10 μm.

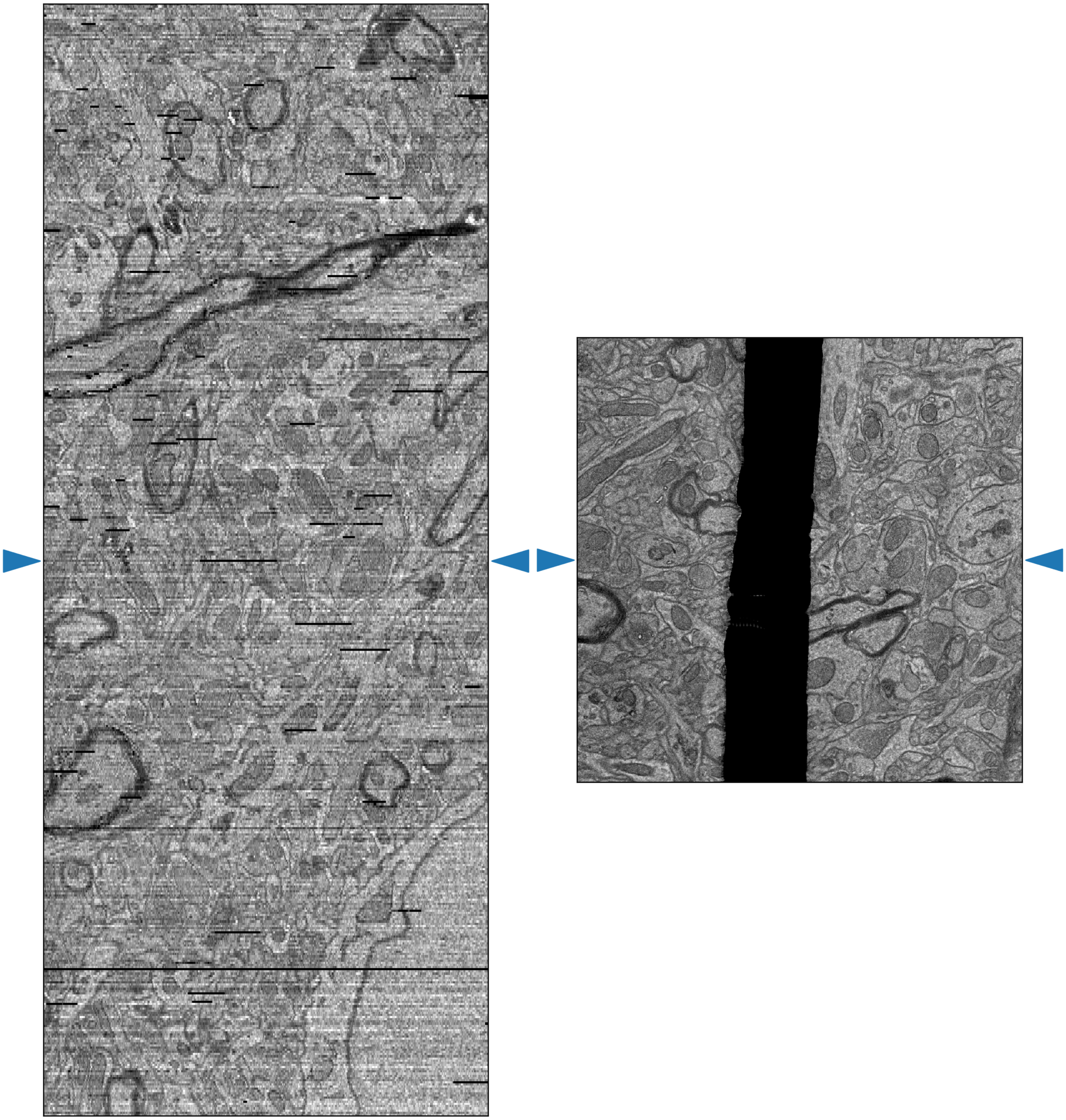

Figure S5. High-resolution view of mouse cortex. From Fig. 3a, 512 sections of mouse cortex displayed orthogonal to the sectioning plane (**a**), and a single in-plane section (**b**). Arrows in one image indicate the plane that was sampled to provide the other image. Folds in the orthogonal view present as horizontal black lines, and there are on the order of one hundred within this field of view. Width of both images is 4 μm.

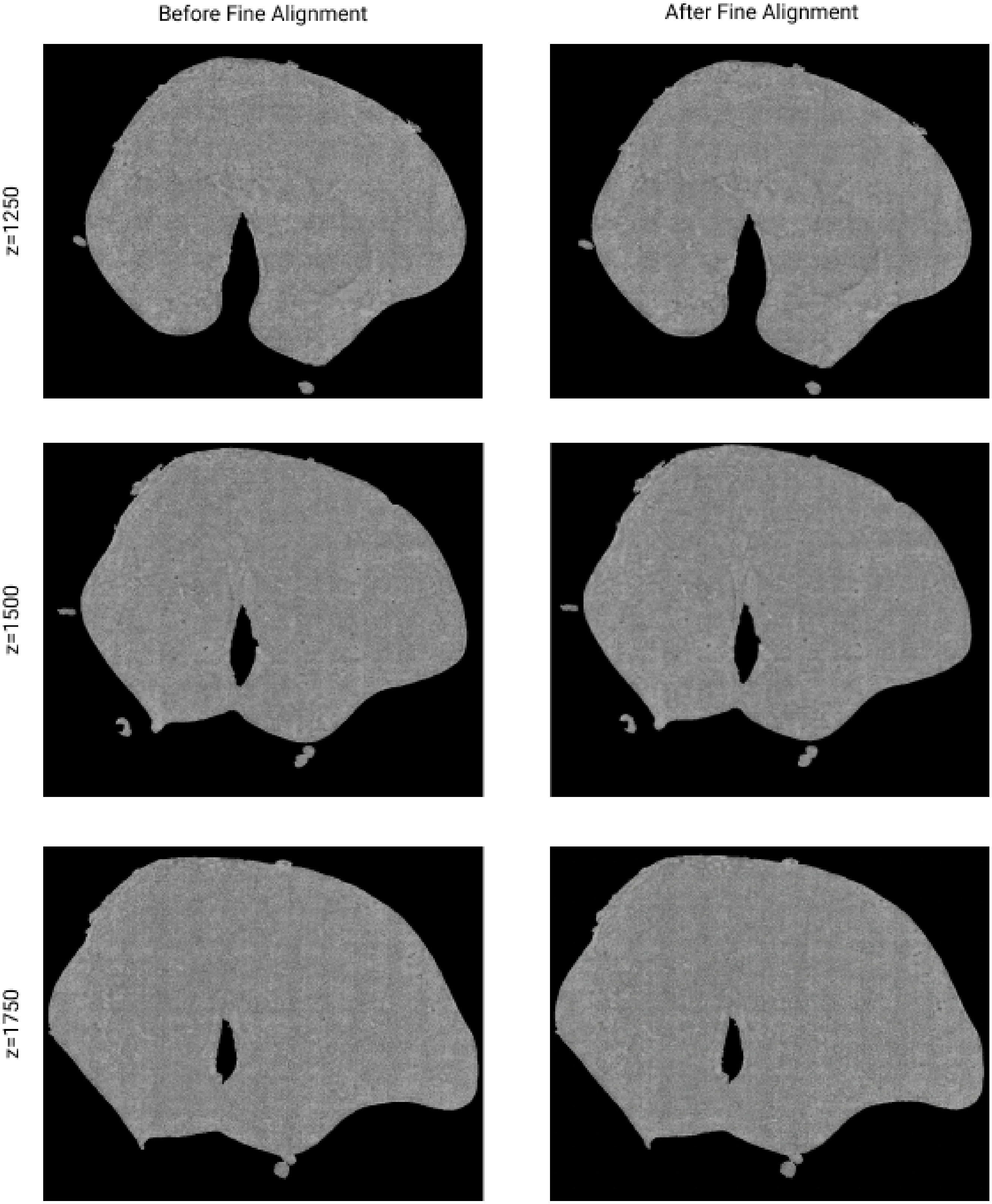

Figure S6. Full section view before and after v15 fine alignment. Width of each image is 400 μm.

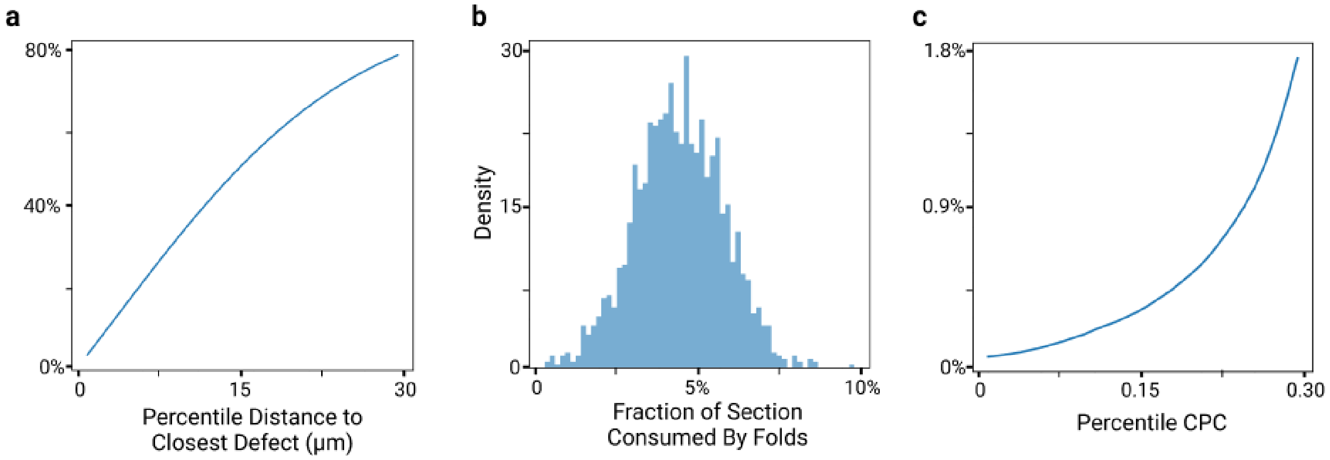

Figure S7. Mouse cortex alignment statistics. Alignment statistics collected over 500 sections of 0.4 x 0.4 mm cutout. The cutout was aligned with our v15 pipeline without Vector Voting. **a**, Percentile plot for the distance of tissue to the closest defect. Defect locations were identified with a dedicated ConvNet. More than 50% of the tissue lies within 20 μm of the fold. **b**, Per-section distribution of the fraction of tissue consumed by folds. Fold locations were identified with a dedicated ConvNet. On average, 4.6% was consumed. **c**, Percentile CPC plot. CPC is performed on chunks of 2048 x 2048 nm at 64 x 64 nm pixel resolution. Non-tissue chunks, chunks inside blood vessels, cell bodies, and chunks that include a discontinuous artifact were excluded.
