# Supplementary material for: Petascale pipeline for precise alignment of images from serial section electron microscopy
Source: Nat Commun. 2024 Jan 4;15:289. doi: 10.1038/s41467-023-44354-0 (PMC10767115; doi:10.1038/s41467-023-44354-0)
Supplement: Supplementary file 2 — Reporting Summary [file 41467_2023_44354_MOESM2_ESM.pdf]

nature portfolio

Corresponding author(s): H. Sebastian Seung

Last updated by author(s): December 6, 2023

Reporting Summary

Nature Portfolio wishes to improve the reproducibility of the work that we publish. This form provides structure for consistency and transparency in reporting. For further information on Nature Portfolio policies, see our Editorial Policies and the Editorial Policy Checklist.

Statistics

For all statistical analyses, confirm that the following items are present in the figure legend, table legend, main text, or Methods section.

n/a	Confirmed	
[ ]	[x]	The exact sample size (*n*) for each experimental group/condition, given as a discrete number and unit of measurement
[ ]	[x]	A statement on whether measurements were taken from distinct samples or whether the same sample was measured repeatedly
[x]	[ ]	The statistical test(s) used AND whether they are one- or two-sided<br>*Only common tests should be described solely by name; describe more complex techniques in the Methods section.*
[x]	[ ]	A description of all covariates tested
[x]	[ ]	A description of any assumptions or corrections, such as tests of normality and adjustment for multiple comparisons
[ ]	[x]	A full description of the statistical parameters including central tendency (e.g. means) or other basic estimates (e.g. regression coefficient) AND variation (e.g. standard deviation) or associated estimates of uncertainty (e.g. confidence intervals)
[x]	[ ]	For null hypothesis testing, the test statistic (e.g. *F*, *t*, *r*) with confidence intervals, effect sizes, degrees of freedom and *P* value noted<br>*Give P values as exact values whenever suitable.*
[x]	[ ]	For Bayesian analysis, information on the choice of priors and Markov chain Monte Carlo settings
[x]	[ ]	For hierarchical and complex designs, identification of the appropriate level for tests and full reporting of outcomes
[x]	[ ]	Estimates of effect sizes (e.g. Cohen's *d*, Pearson's *r*), indicating how they were calculated

Our web collection on statistics for biologists contains articles on many of the points above.

Software and code

Policy information about availability of computer code

Data collection	Data must be accessible via CloudVolume>=8.19.3<br>https://github.com/seung-lab/cloud-volume
Data analysis	Python 3.7, 3.8, 3.9<br>Corgie, https://github.com/seung-lab/corgie<br>MetroEM, https://github.com/seung-lab/metroem<br>EM_Aligner montage diagnostic tool, https://bit.ly/3gI5Cg0<br>BigFeta, https://github.com/AllenInstitute/BigFeta<br>Iterative TrakEM2 solver client, https://bit.ly/3gCuu98

For manuscripts utilizing custom algorithms or software that are central to the research but not yet described in published literature, software must be made available to editors and reviewers. We strongly encourage code deposition in a community repository (e.g. GitHub). See the Nature Portfolio guidelines for submitting code & software for further information.

Data

Policy information about availability of data

All manuscripts must include a data availability statement. This statement should provide the following information, where applicable:

- Accession codes, unique identifiers, or web links for publicly available datasets
- A description of any restrictions on data availability
- For clinical datasets or third party data, please ensure that the statement adheres to our policy

FAFB v15 data is publicly available from https://seung-lab.github.io/fafbv15/. The FAFB v14.1 dataset is hosted by BossDB 40 at https://bossdb.org/project/flywire. The two mouse cortex volumes are also hosted by BossDB at https://bossdb.org/project/microns-minnie and https://bossdb.org/project/microns-interneuron.

Research involving human participants, their data, or biological material

Policy information about studies with human participants or human data. See also policy information about sex, gender (identity/presentation), and sexual orientation and race, ethnicity and racism.

Reporting on sex and gender	Not applicable
Reporting on race, ethnicity, or other socially relevant groupings	Not applicable
Population characteristics	Not applicable
Recruitment	Not applicable
Ethics oversight	Not applicable

Note that full information on the approval of the study protocol must also be provided in the manuscript.

Field-specific reporting

Please select the one below that is the best fit for your research. If you are not sure, read the appropriate sections before making your selection.

☒ Life sciences ☐ Behavioural & social sciences ☐ Ecological, evolutionary & environmental sciences

For a reference copy of the document with all sections, see nature.com/documents/nr-reporting-summary-flat.pdf

Life sciences study design

All studies must disclose on these points even when the disclosure is negative.

Sample size	Using the Chunked Pearson Correlation (CPC), we evaluated the alignment of 1000 sections of FAFB, which represents more than 14% of the entire dataset. When estimating the number of misaligned locations in this 1000 sections, we inspected 300 regions (2 x 2 um^2) with low CPC scores to manually classify if a misalignment was present. The sample size of 300 was chosen because it could be evaluated in under an hour for each test, and provided significant margin for low confidence intervals.
Data exclusions	In reporting aggregate statistics of the CPC, we excluded 2 x 2 um^2 regions containing either a crack or fold in the data (locations that had been predetermined), or regions that did not contain tissue, where CPC could not accurately indicate a misalignment.
Replication	We quantitatively evaluated the quality of our alignment pipeline over alternative alignments using data from an adult fly brain. We also used our pipeline to align data from mouse cortex, where we qualitatively evaluated comparable quality.
Randomization	There was no randomization between class labels.
Blinding	Manual annotators were not blind to the conditions used to produce the evaluated data. The presence of a misalignment can be classified with high confidence, so the impact of bias seemed low.

Reporting for specific materials, systems and methods

We require information from authors about some types of materials, experimental systems and methods used in many studies. Here, indicate whether each material, system or method listed is relevant to your study. If you are not sure if a list item applies to your research, read the appropriate section before selecting a response.

Materials & experimental systems

n/a	Involved in the study
☒	☐ Antibodies
☒	☐ Eukaryotic cell lines
☒	☐ Palaeontology and archaeology
☒	☐ Animals and other organisms
☒	☐ Clinical data
☒	☐ Dual use research of concern
☒	☐ Plants

Methods

n/a	Involved in the study
☒	☐ ChIP-seq
☒	☐ Flow cytometry
☒	☐ MRI-based neuroimaging
